# Distinct myofibrillar sub-proteomic profiles are associated with the instrumental texture of aged pork loin

**DOI:** 10.1093/jas/skad327

**Published:** 2023-09-26

**Authors:** Logan G Johnson, Chaoyu Zhai, Edward M Steadham, Leah M Reever, Kenneth J Prusa, Mahesh N Nair, Elisabeth Huff-Lonergan, Steven M Lonergan

**Affiliations:** Department of Animal Science, Iowa State University, Ames, IA 50011, USA; Department of Animal Science, University of Connecticut, Storrs, CT 06269-4040, USA; Department of Animal Science, Iowa State University, Ames, IA 50011, USA; Department of Food Science and Human Nutrition, Iowa State University, Ames, IA 50011, USA; Department of Food Science and Human Nutrition, Iowa State University, Ames, IA 50011, USA; Department of Animal Sciences, Colorado State University, Fort Collins, CO 80523, USA; Department of Animal Science, Iowa State University, Ames, IA 50011, USA; Department of Animal Science, Iowa State University, Ames, IA 50011, USA

**Keywords:** liquid chromatography-mass spectrometry, myofibrillar, pork, protein fractionation, proteomics, tandem mass tag

## Abstract

Fresh pork tenderness contributes to consumer satisfaction with the eating experience. Postmortem proteolysis of proteins within and between myofibrils has been closely linked with pork tenderness development. A clear understanding of the molecular features associated with pork tenderness development will provide additional targets and open the door to new solutions to improve and make pork tenderness development more consistent. Therefore, the objective was to utilize liquid chromatography and mass spectrometry with tandem mass tag (TMT) multiplexing to evaluate myofibrillar sub-proteome differences between pork chops of different instrumental star probe values. Pork loins (*N* = 120) were collected from a commercial harvest facility at 24 h postmortem. Quality and sensory attributes were evaluated at 24 h postmortem and after ~2 weeks of postmortem aging. Pork chops were grouped into 4 groups based on instrumental star probe value (group A,$\bar{x}$ = 4.23 kg, 3.43 to 4.55 kg; group B,$\bar{x}$ = 4.79 kg, 4.66 to 5.00 kg; group C,$\bar{x}$ = 5.43 kg, 5.20 to 5.64 kg; group D,$\bar{x}$ = 6.21 kg, 5.70 to 7.41 kg; *n* = 25 per group). Myofibrillar proteins from the samples aged ~2 wk were fractionated, washed, and solubilized in 8.3 M urea, 2 M thiourea, and 1% dithiothreitol. Proteins were digested with trypsin, labeled with 11-plex isobaric TMT reagents, and identified and quantified using a Q-Exactive Mass Spectrometer. Between groups A and D, 54 protein groups were differentially abundant (adjusted *P* < 0.05). Group A had a greater abundance of proteins related to the thick and thin filament and a lesser abundance of Z-line-associated proteins and metabolic enzymes than group D chops. These data highlight that distinct myofibrillar sub-proteomes are associated with pork chops of different tenderness values. Future research should evaluate changes immediately and earlier postmortem to further elucidate myofibrillar sub-proteome differences over the postmortem aging period.

## Teaser

The results highlight the value of fractionating the myofibrillar sub-proteome before liquid chromatography-mass spectrometry analysis. The approach provides more focused and detailed information about the trait of interest. Moreover, it can be deduced that postmortem aging results in very different modifications (notably proteolysis) that are not normally observed in living muscle.

## Introduction

Maintaining and improving pork quality—specifically tenderness—remain important priorities in the pork industry. Tenderness is affected by many factors and events that occur postmortem and post-rigor, making it expensive and difficult to predict. Predicting pork tenderness requires thoroughly understanding the metabolic, biochemical, and composition factors associated with pork tenderness. It is understood that some features, such as lipid content and pH, contribute to ultimate tenderness, but these predictors could be more robust. Aging meat at refrigerated temperatures following rigor completion leads to a notable reduction in shear force and a considerable improvement in the tenderness of meat (Huff Lonergan et al., 2010; Schulte et al., 2019). A substantial body of evidence supports the hypothesis that postmortem protein degradation of myofibrillar proteins contributes to the resolution of rigor and enhanced tenderness of meat (Geesink et al., 2006; Warner et al., 2021). Isolating the myofibrillar fraction before analysis provides a chance to focus on those changes, especially in some proteins that are less ­abundant. Defining the myofibrillar sub-proteome phenotypes in aged meat associated with differences in ultimate tenderness will inform future investigations and efforts to predict ultimate pork tenderness.

In postmortem skeletal muscle, the degradation of specific proteins, including titin, nebulin, desmin, filamin, and troponin-T, has been associated with meat tenderness development (Huff-Lonergan et al., 1996a; Wheeler et al., 2000; Melody et al., 2004; Carlson et al., 2017b). These proteins and others exist primarily within and between myofibrils and are frequently indicators of postmortem proteolysis. These proteins are soluble primarily under higher ionic conditions as intact proteins. Incubation of calpain with purified myofibrils results in the release of intact alpha-actinin to the supernatant (Goll et al., 1991), and the aged pork sarcoplasmic sub-proteome contains the rod portion desmin (Carlson et al., 2017a). Postmortem proteolysis, therefore, influences the solubility of proteins and peptides, some of which are related to tenderness development and contribute to changes in the postmortem myofibrillar sub-proteome.

Previous studies have utilized 1-dimensional sodium dodecyl sulfate-polyacrylamide gel (SDS-PAGE) or 2-dimensional difference in gel electrophoresis methods coupled with mass spectrometry to identify and quantify specific proteins related to pork quality attributes (Hwang et al., 2005; Carlson et al., 2017a; López-Pedrouso et al., 2020; Schulte et al., 2020; Zuber et al., 2021). Gel-free methods utilizing tryptic digestion and liquid chromatography–tandem mass spectrometry (LC–MS/MS) analysis with isobaric tags or label-free techniques have recently been applied to beef (Schulte et al., 2023) and pork samples (Huang et al., 2018; Liu et al., 2021, 2022; Zequan et al., 2021; Johnson et al., 2023). Few of these studies have aimed to specifically evaluate the extent to which differences within the myofibrillar sub-proteome are associated with meat quality. Therefore, this study aimed to determine the differences within the myofibrillar sub-proteome from aged pork loins classified by instrumental star probe value using LC–MS/MS approaches. It was hypothesized that aged pork chops with a lower instrumental star probe value would have more postmortem proteolysis, resulting in a greater release of soluble peptides and protein fragments from intermediate filament and structural proteins from the aged myofibrillar sub-proteome than pork chops with a higher instrumental star probe value.

## Materials and Methods

### Pork loin quality and sensory data collection

The current study was conducted only on pork loins collected from a commercial pork harvest facility following standard humane slaughter practices according to USDA guidelines; therefore, Institutional Animal Care and Use approval was not sought. The population of pork loins (*N* = 120) was collected from a commercial pork harvest facility at 1 d postmortem on three separate collection days over 3 wk. The pH at 1 d postmortem was recorded during the loin collection using a Hanna HI 9025 pH meter (Hanna Instruments, Woonsocket, RI). The loins were vacuum packaged, transported on ice to Iowa State University, and aged for 12 or 14 d postmortem at 4 °C, where aging time varied to avoid freezing samples before sensory and quality analysis ­(Carlson et al., 2017b). Fresh pork loin quality measurements, a trained sensory panel, and moisture and lipid proximate data were collected and analyzed as described by Johnson et al. (2023). The range and standard deviation of the quality and sensory measurements for the initial population of pork loins (*N = *120) are reported in Supplementary Table S1. The aged pork loin chops were classified based on instrumental star probe measurements into 4 groups (group A,$\bar{x}$ = 4.23 kg, 3.43 to 4.55 kg; group B,$\bar{x}$ = 4.79 kg, 4.66 to 5.00 kg; group C,$\bar{x}$ = 5.43 kg, 5.20 to 5.64 kg; group D,$\bar{x}$ = 6.21 kg, 5.70 to 7.41 kg; *n* = 25 per group). The quality phenotype of each star probe group is summarized in Table 1. The subset of pork chops (*n *= 100) was from a relatively equal distribution of barrows (*n *= 53) and gilts (*n* = 47) from three sire lines (line 1, *n* = 34; line 2, *n* = 33; line 3, *n* = 33) collected across three slaughter dates (date 1, *n* = 32; date 2, *n* = 36; date 3, *n* = 32).

**Table 1.** Summary of pork quality and sensory attributes from aged pork loins classified by instrumental star probe group

Attribute	Group A* (*n* = 25)	Group B* (*n* = 25)	Group C* (*n* = 25)	Group D* (*n* = 25)	SEM	Star probe group *P*-value	Sex *P-*value	Sire line *P*-value	Slaughter date *P*-value
Star probe, kg^1^	4.23^d^	4.79^c^	5.43^b^	6.21^a^	0.06	<0.001	0.003	0.25	0.96
24 h pH	5.75^a^	5.7^ab^	5.65^bc^	5.63^c^	0.02	<0.001	0.36	0.07	0.05
Aged pH	5.84^a^	5.79^b^	5.75^bc^	5.73^c^	0.02	<0.001	0.32	0.002	0.90
Loin purge, %	0.32^c^	0.5^bc^	0.9^ab^	0.75^a^	0.12	0.004	0.51	0.06	0.13
Chop purge, %	0.58	0.73	0.83	0.83	0.07	0.06	0.81	0.02	0.37
Moisture content, %	74.71	74.68	74.81	74.81	0.18	0.92	0.12	0.03	0.44
Lipid content, %	2.39^a^	1.96^b^	1.96^b^	1.86^b^	0.14	0.07	0.02	0.004	0.63
Marbling score^2^	2.4^a^	2.0^b^	2.1^b^	1.8^b^	0.1	0.01	0.005	0.009	0.10
Color score^3^	3.5^a^	3.2^ab^	3.1^bc^	2.9^c^	0.1	0.009	0.03	0.01	0.07
Aged L*^4^	47.74^b^	47.66^ab^	48.20^a^	49.32^a^	0.43	0.045	0.97	0.02	0.03
Cook loss, %	21.26^b^	22.34^b^	22.87^ab^	23.54^a^	0.53	0.036	0.76	0.84	0.07
Tenderness^5^	7.6^a^	7.1^a^	6.4^b^	6.3^b^	0.2	<0.001	0.12	0.92	0.002
Chewiness^5^	2.5^c^	3.1^b^	3.5^ab^	3.9^a^	0.2	<0.001	0.62	0.10	0.01
Juiciness^5^	6.8^a^	6.7^ab^	6.4^ab^	6.2^b^	0.2	0.13	0.36	0.55	0.01
Flavor^5^	5.5^a^	4.5^b^	4.2^b^	3.6^c^	0.2	<0.001	0.78	0.06	0.10
Off flavor^5^	1.3^b^	1.5^b^	1.7^b^	2.3^a^	0.1	<0.001	0.59	0.045	0.10
55 kDa intact desmin^6^	0.47^b^	—	—	0.94^a^	0.05	<0.001	—	—	—

^1^A 5-point star probe attachment fitted with an Instron was used to assess the force needed to compress a chop to 20% of its original height (Carlson et al., 2017b).

^2^National Pork Board standards, 10-point scale (1 = 1% intramuscular fat; 10 = 10% intramuscular fat).

^3^National Pork Board standards, 6-point scale (1 = pale pinkish gray/white; 6 = dark purplish red).

^4^Hunter L* determined with Minolta Chroma Meter with D65 light source, 50 mm aperture, and 0° observer.

^5^As determined by a trained panel (*N* = 4) using a 10-point category scale.

^6^Ratio of the densitometry units of the intact 55 kDa desmin band of the sample over the intact 55 kDa desmin band of the reference sample.

^a,b,c,d^Means within rows with different superscripts are significantly different (*P* < 0.05).

*From the distribution of star probe values of the initial population of pork loins (*N *= 120), chops in group A represent the lowest 0 to 20% in value, group B is the next highest 25 to 45%, group C is the next 55 to 75%, and group D is the highest 80 to 100%.

### Myofibrillar protein extraction

Myofibrillar proteins from the pork chops (*n* = 100) classified into the star probe groups were prepared individually. Frozen pork loin (frozen after aging 12 or 14 d postmortem) containing only the longissimus dorsi (LD) muscle (~200 g) was homogenized and uniformly powdered in liquid nitrogen. Powdered samples were stored at −80 °C until protein extraction. Myofibrillar proteins were extracted according to Carlson et al. (2017a, 2017b) with minor modifications. Approximately 1.5 g of powdered sample was homogenized with 4.5 mL of ice-cold low-ionic strength buffer [50 mM Tris–HCl (pH 8.5) and 1 mM ethylenediaminetetraacetic acid (EDTA)] using a Polytron PT 3100 (Polytron, Lucerne, Switzerland). Each sample was homogenized in two 10-s bursts and kept on ice. Homogenates were centrifuged at 24,446 × *g* for 30 min at 4 °C, and the supernatant was discarded. The insoluble pellet weight was recorded, and 20 mL of standard salt solution [100 mM potassium chloride, 20 mM potassium phosphate, 2 mM magnesium chloride, 2 mM ethylenebis(oxyethylenenitroilo)tetraacetic acid (EGTA), and 1 mM sodium azide] was added to the insoluble pellet. The pellet was homogenized briefly (less than 5 sec) using a Polytron PT 3100 (Polytron) and collected at 1,000 × *g* for 10 min at 4 °C. After the supernatant was discarded, 20 mL of standard salt solution was added to the pellet and then vortexed for 10 s to break apart the pellet. The pellets were washed with standard salt solution and collected with an identical centrifugation three times. The pellet was washed with 25 mL of Tris buffer [5 mM Tris–HCl (pH 8.0)] by vortex mixing for 10 s and collected at 3,020 × *g* for 10 min at 4 °C. The supernatant was discarded, and the pellet was washed with 25 mL of Tris buffer by vortex mixing for 10 s and collected at 24,446 × *g* for 10 min at 4 °C. From the washed pellet, myofibrillar proteins were extracted by adding 5 vol (based on the initial pellet weight) of myofibrillar extraction buffer [8.3 M urea, 2 M thiourea, and 1% (v/v) dithiothreitol] and adjusted to pH 8.5 with 2 M Tris–HCl (pH 8.8). The pellet was vortexed for 10 s and placed on a rocker for 30 min at 4 °C. The samples were centrifuged at 24,446 × *g* for 15 min at 4 °C, transferred to a clean tube, and clarified at 24,446 × *g* for 15 min at 4 °C. The protein concentration of the supernatant was determined using Bradford QuickStart reagents (Bio-Rad Laboratories, Hercules, CA). All samples were adjusted to 8 mg/mL with myofibrillar extraction buffer, vortexed, and stored at −80 °C. Separately, an aliquot from each sample was adjusted to 6.4 mg/mL with myofibrillar extraction buffer and further diluted to 4 mg/mL with 0.5 volume of protein denaturing buffer [3 mM EDTA, 3% (w/v) sodium dodecyl sulfate (SDS), 30% (v/v) glycerol, 0.001% (w/v) pyronin Y, and 30 mM Tris–HCl (pH 8.0)] and 0.1 volume of 2-mercaptoethanol (Huff-Lonergan et al., 1996b). Samples were vortexed, heated on a dry heat block for 15 min at ~50 °C, and stored at −80 °C. Confirmation of equal protein concentrations between samples was evaluated using 15% SDS–PAGE with a 5% stacking gel with 10 lanes, as described by Johnson et al. (2023).

### Western blotting

The prepared 4 mg/mL myofibrillar extracts from aged pork chops classified as group A (*n* = 25) and group D (*n* = 25) were used to evaluate desmin with Western blotting using one-dimensional SDS-PAGE. A pooled reference was prepared by mixing equal volumes of 10 samples, 5 samples each from groups A and D, and included on each gel. SDS–PAGE gels (15%) with a 5% stacking gel with 10 lanes were prepared. The first lane was loaded with Precision Plus Protein All Blue molecular weight standards (Bio-Rad). The remaining lanes were loaded with 40 µg of protein. Proteins were resolved at 130 V for ~360 V/h in Hoefer 260 Mighty Small II units (Hoefer, Inc., Holliston, MA). Following electrophoresis, proteins were transferred to polyvinylidene difluoride (PVDF) membranes (0.2-μm pore size) using TE-22 Mighty Small Transphor units (Hoefer, Inc.), running for a constant voltage of 90 V for 90 min at ~5 °C. The transfer buffer contained 25 mM Tris, 192 mM glycine, and 15% (v/v) methanol.

**Figure 1.**
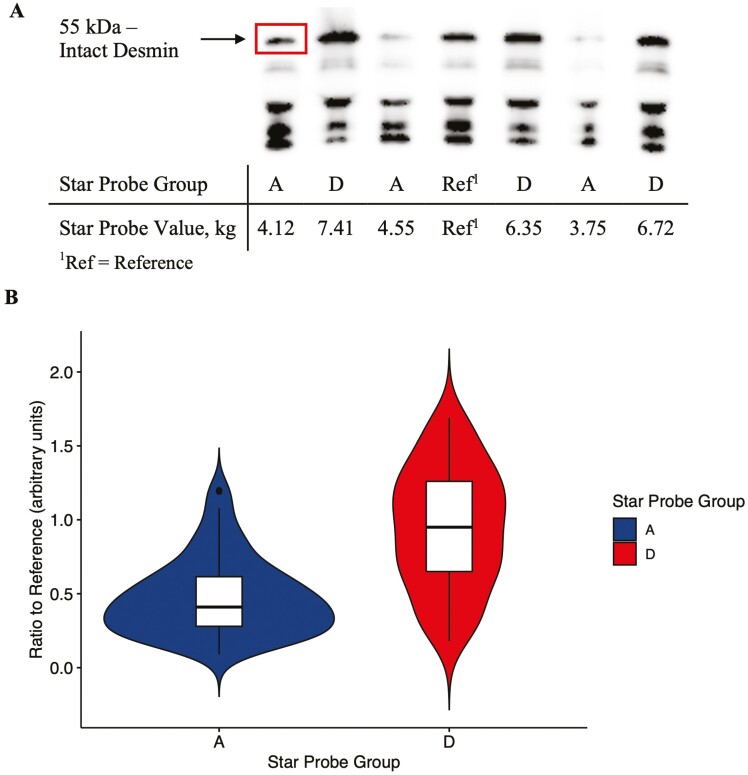
(A) Representative Western blot of desmin from the aged myofibrillar extract of pork chops classified as groups A and D by instrumental star probe. Intact desmin (55 kDa) bands were compared to the 55 kDa intact band from the reference sample included on all gels. The reference was a mixture of an equal amount of protein from myofibrillar samples classified as groups A and D. (B) Violin plot of 55 kDa intact desmin ratios to the reference for all technical replicate samples. Bottom border, interior line, and top border of the boxplot represent the 1st quartile, median, and 3rd quartile, respectively.

After transfer, membranes were blocked for 1 h in PBS-Tween [80 mM Na_2_HPO_4_, 20 mM NaH_2_PO_4_, 100 mM NaCl, and 0.1% (v/v) polyoxyethylene sorbitan ­monolaurate (Tween 20)] containing 5% (wt./vol) nonfat dry milk. Membranes were then incubated overnight with polyclonal rabbit anti-desmin antibody produced at Iowa State University (Huff-Lonergan et al., 1996a, 1996b) diluted 1:40,000 in PBS-Tween buffer. Membranes were washed with PBS-Tween buffer 3 times for 10 min each. The secondary goat anti-rabbit-HRP antibody (31460; Thermo Scientific) was diluted 1:15,000 in PBS-Tween buffer, and membranes were incubated with the secondary antibody for 1 h at room temperature. Membranes were subsequently washed with PBS-Tween buffer 3 times for 10 min each. Proteins were detected using a chemiluminescent detection kit (ECL Prime; GE Healthcare, Piscataway, NJ), and images of blots were obtained and analyzed using a ChemiImager 5500 (Alpha Innotech, San Leandro, CA) and Alpha Ease FC software (version 3.03; Alpha Innotech). Densitometry was used to quantify the intact 55-kDa desmin band, and comparisons were made by taking the ratio of the measured protein band to the internal reference. At least two technical replicates of each sample (*n *= 50) were conducted on separate days, and a representative Western blot is included in Figure 1A.

### Tandem mass tag analysis

The prepared myofibrillar extracts from each aged pork chop grouped by instrumental star probe (*n* = 100) were submitted to the Iowa State Protein Facility for tandem mass tag (TMT) analysis, where the peptide preparation, chromatographic separation, and ion detection specifications were described previously (Johnson et al., 2023). Briefly, 5 µg from each ­sample was pooled, reduced, alkylated, and digested with trypsin according to the manufacturer’s directions to serve as the Master Control sample. The peptide concentration was determined using a Pierce Colorimetric kit (ThermoFisher), and the Master Control peptides were labeled with the TMT11-131C reagent (A37724, Thermo Scientific).

Individually, each sample was reduced, alkylated, and digested with trypsin according to the manufacturer’s directions. Samples were randomly assigned to 1 of 10 different LC–MS/MS runs and 1 of 10 different TMT tags within a run. Samples were subsequently balanced to achieve a near-equal distribution of star probe groups within and across the 10 LC–MS/MS runs. The peptide concentration was quantified using a Pierce Colorimetric kit (Thermo Scientific). Each sample (25 µg) was incubated with 0.2 mg of a TMT10plex Label Reagents (90110, Thermo Scientific) for 1 h. The digested and labeled peptides from each sample (3 µg) within a run and the master control (3 µg) were pooled (33 µg total) and dried using a Savant SpeedVac Plus (Thermo Scientific). The pooled runs were reconstituted with 33 µL of 5% acetonitrile and 0.1% trifluoroacetic acid.

Chromatographic separation of peptides was achieved using an EASY-nLC 1200 (Thermo Scientific) system with an integrated autosampler. The column was equilibrated with Buffer A (0.1% formic acid in water). Peptides were eluted and introduced into the Q Exactive Hybrid Quadrupole-Orbitrap Mass Spectrometer (Thermo Scientific) with a Higher Energy Collisional Dissociation (HCD) cell with a Nanospray Flex ion source (Thermo Scientific). The elution gradient consisted of a linear gradient of 0% to 35% Buffer B (0.1% formic acid in acetonitrile) over 240 min, a linear gradient of 35% to 70% Buffer B over 20 min, and a linear gradient of 70% to 100% Buffer B over 4 min at a flow rate of 300 nL/min. Samples were analyzed during MS1 with an automatic gain control (AGC) target of 1 × 10^6^ and a maximum injection time of 80 ms. The MS1 scans were analyzed at 70,000 resolving power, and precursor ions were selected within a scan range of 400 to 2,000 *m/z* during positive ionization mode. The MS2 scans were collected with an isolation window of 1.2 *m*/*z* and fragmented at a 32% normalized collision energy. The ions were analyzed at 35,000 resolving power with an AGC target of 1 × 10^5^ and a maximum injection time of 50 ms. Spectral data were processed in Proteome Discoverer (version 2.5.0.400; Thermo Scientific), with search parameters and peptide and protein matching described previously (Johnson et al., 2023).

### Statistical analysis

Pork quality and sensory attributes and intact desmin data were analyzed using R (version 4.2.2) and RStudio. A one-way analysis of variance was used with the fixed effect of star probe group. Estimated marginal means were computed using the *emmeans* (v. 1.8.5) package to make pairwise comparisons between star probe groups. The fixed effects of sex, harvest date, and sire line were tested separately for each attribute and were reported in Table 1. Significance was denoted by *P *< 0.05, and a trend was denoted by *P* < 0.1.

The reporter ion intensities were normalized to the total ion count within each run. The ratio of each TMT10-plex reporter ion abundance relative to the Master Control within a run for an identified protein was calculated. Eight individual samples were removed from the analysis due to low peptide detection within the sample. Only proteins identified in at least half of the samples (*n* ≥ 46) containing at least 2 unique peptides were retained. The ratios were Log_2_ transformed and normalized to the median value within a sample. Statistical analysis was conducted using R and RStudio using the *limma* (v. 3.54.2) package (Ritchie et al., 2015), where moderated *t*-tests were used to make pairwise comparisons between star probe groups. The Benjamini-Hochberg adjustment method was used at 5% to control the FDR of differentially abundant proteins between star probe groups. Proteins were considered differentially abundant at an adjusted *P *< 0.05.

## Results and Discussion

There were significant differences in pork quality across the categories defined based on instrumental star probe measurements (Table 1). Pork chops in group A had an average star probe value 2 kg lower than those in group D. These differences in tenderness were also identified by trained sensory panelists, where Group A chops were rated as more tender (*P *< 0.05) and less chewy (*P *< 0.05) than Group D. Other quality and sensory traits were affected by classification, where Group A chops had more pork flavor (*P *< 0.05) and less off-flavor (*P* < 0.05) than chops in Group D. Group A chops had a higher pH at 24 h (*P* < 0.05) and after aging (*P* < 0.05), less loin purge loss (*P* < 0.05), less cook loss (*P* < 0.05), and tended to have less chop purge (*P* = 0.06) than Group D chops. There were no differences in moisture content between the groups (*P* > 0.05). Visual marbling scores were greater (*P* < 0.05), and lipid content tended (*P* = 0.07) to be greater in Group A chops, while visual color scores were greater (*P* < 0.05) and aged L* values were lower (*P* < 0.05) in Group A compared to Group D chops.

Overall, a lower star probe value resulted in pork chops with a higher pH, less purge and cook loss, a lower L*, and greater sensory measures. Additionally, as the difference in star probe value between groups increased, generally, the more divergent in other quality attributes and the magnitude of those differences increased. For example, aged pH was higher (*P* < 0.05) in Group A than in Group B and C, and higher (*P* < 0.05) in Group B than D, visual color scores were greater (*P* < 0.05), and aged L* values were lower (*P* < 0.05) in Group A vs. Groups C and D, and chewiness scores were lower (*P* < 0.05) and flavor scores were higher (*P* < 0.05) in Group A vs. Group B and similarly between Group B vs. Group D. Therefore, this pork loin population is ideal for investigating specific proteome differences in the aged myofibrillar sub-proteome to better understand the biochemical changes associated with meat tenderness development.

**Figure 2.**
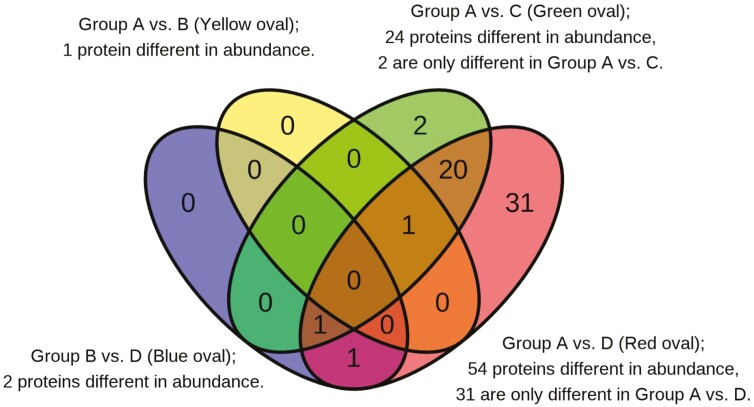
The number of significantly different proteins between star probe groups. The number of significantly different proteins shared between pairwise comparisons of star probe groups. Overlapping color regions indicate the number of proteins that were similarly different between pairwise comparisons. Refer to Tables 2 and 3 for a list of the proteins different between comparisons.

In the current study, 427 protein groups were identified in at least one of the ten LC–MS/MS runs after spectral, peptide, and protein matching. After filtering proteins that contained 2 unique peptides or greater and were present in at least half of the samples (*n* ≥ 46), 202 protein groups were retained and included in the analysis. Figure 2 shows the number of significantly different proteins between pairwise comparisons of star probe groups. Notably, the aged myofibrillar sub-proteome was more divergent between the groups with greater differences in tenderness traits. This observation provides confidence that the proteomic and pork quality phenotypes are related. Table 2 includes the differentially abundant proteins and the Log_2_ fold difference between any pairwise comparison of star probe groups but does not include all differentially abundant proteins between star probe groups A and D. Table 2 demonstrates that as the pork quality phenotype becomes increasingly divergent in instrumental star probe value, the number of differentially abundant proteins increases, and generally the magnitude of Log_2_ fold difference in abundance also increases. For example, only 1 protein was differentially abundant between groups A and B, and groups A and B had minimal differences in measured quality and sensory attributes. Between groups A and C and groups A and D, however, the number of differentially abundant proteins increased along with a greater divergence in quality. Table 3 shows the 54 differentially abundant proteins between the most divergent star probe groups A and D. A brief overview of proteins not significantly different (*P* ≥ 0.05) between groups A and D is presented in Supplementary Table S2. Utilizing the literature to aid in assigning function to the differentially abundant proteins, broad groups of proteins are identified, including those that are primarily thick and thin filament, Z-line, structural, and metabolic proteins. The remaining discussion will focus on the differences between groups A and D chops, as they are the most phenotypically divergent on a loin quality and myofibrillar sub-proteome level.

**Table 2.** Summary of proteins in the aged myofibrillar sub-proteome from pork chops that were differentially abundant (adjusted *P*-value < 0.05) in pairwise comparisons of group A vs. B, B vs. D, A vs. C, and A vs. D

Protein description	Accession number^1^	Log_2_ fold difference^2^			
		A/B	B/D	A/C	A/D
Four and a half LIM domains protein 1 isoform 5 (FHL1)	F6PXR6	−0.46		−0.64	−0.80
Nebulin isoform 3	A0A480SKX8			−0.71	−0.76
PDZ and LIM domain protein 7 (PDLIM7)^3^	A0A4X1SEG0			−0.57	−0.74
α-1,4 glucan phosphorylase	A0A4X1VBN9		−0.43		−0.71
Glyceraldehyde-3-phosphate dehydrogenase	P00355			−0.73	−0.69
Glyceraldehyde-3-phosphate dehydrogenase (fragment)	G3CKJ2			−0.67	−0.69
LIM domain-binding protein 3 (LDB3)^3^	A0A287A435			−0.58	−0.68
Myozenin-1	Q4PS85		−0.35	−0.44	−0.58
PDZ and LIM domain protein 5 (PDLIM5)	A0A287BI36			−0.39	−0.40
WD repeat domain 1^3^	A0A4X1SGJ0			−0.41	−0.36
Titin^3^	A0A4X1U902			−0.27	−0.35
Titin^3^	A0A5G2QM05			−0.25	−0.35
Heat shock protein β 6 (HSPB6)^3^	A0A4X1TIY7			−0.49	−0.30
Heat shock protein β 1 (HSPB1)^3^	A0A5S6G3Y8			−0.32	−0.26
Myotilin^3^	A0A4X1V5J3			−0.27	−0.25
α-Actinin 2	F1RHL9			−0.19	−0.25
Obscurin^3^	A0A5G2QZ79			−0.19	−0.23
Elongation factor 1-α	A0A4X1SRH0			−0.18	−0.21
Desmin	P02540			−0.23	−0.19
Myosin-4	Q9TV62			0.15	0.14
Myosin light chain 1^3^	A0A4X1UTR8			0.16	0.16
Tropomodulin 4^3^	A0A4X1VVT7			0.15	0.19
Peptidase S1 domain-containing protein	A0A4X1V2S2			0.19	0.20
Filamin C	F1SMN5			−0.14	
F-actin-capping protein subunit β	A0A5K1U188			0.11	

^1^Accession number = Uniprot accession number.

^2^Log_2_ fold difference = numerator/denominator; positive number = greater in numerator vs. denominator, negative number = lesser numerator vs. denominator.

^3^These proteins were initially labelled “Uncharacterized” or were labelled with a less commonly identified name. The FASTA sequence of the protein was used to match each protein to a more commonly recognized or accepted name using the UniProt BLAST feature.

**Table 3.** Summary of proteins in the aged myofibrillar sub-proteome that were differentially abundant between groups A and D pork chops

Protein description	Accession number^1^	Subcellular location	Sequence coverage^2^	Unique peptides	Log_2_ fold difference^3^	Adjusted *P*-value
*Thick and thin filament*						
Actin, α skeletal muscle	A0A481CYB2	Thin filament	68	4	0.146	0.009
Myosin binding protein C, fast type^4^	A0A4X1VUZ8	Thick filament	66	67	0.120	0.036
Myosin binding protein H	I3LIE7	Thick filament	70	20	0.201	0.011
Myosin light chain 1^4^	A0A4X1UTR8	Thick filament	88	2	0.160	0.004
Myosin regulatory light chain 2 (RLC-2)^4^	A0A4X1TZM9	Thick filament	84	16	0.167	0.018
Myosin regulatory light polypeptide 9	A0A4X1TGS8	Thick filament	23	3	0.550	0.040
Myosin-1	Q9TV61	Thick filament	66	11	0.268	0.029
Myosin-4	Q9TV62	Thick filament	69	6	0.139	0.008
Myosin-4	A0A5G2RAA2	Thick filament	69	2	0.175	0.024
Tropomodulin 4^4^	A0A4X1VVT7	Thin filament	62	13	0.185	0.002
Troponin T, fast skeletal muscle	A0A4X1UDR6	Thin filament	31	12	0.221	0.022
*Z-line*						
Alpha-actinin 2	F1RHL9	Z-line	66	35	-0.245	< 0.001
Four and a half LIM domains protein 1 isoform 5 (FHL1)	F6PXR6	Z-line/M-line	65	18	-0.802	< 0.001
LIM domain-binding protein 3 (LDB3)^4^	A0A287A435	Z-line	33	14	-0.680	< 0.001
Myotilin^4^	A0A4X1V5J3	Z-line	42	16	-0.248	0.009
Myozenin-1	Q4PS85	Z-line	76	14	-0.582	< 0.001
PDZ and LIM domain protein 3 (PDLIM3)	A0A4X1VY45	Z-line	21	4	-0.531	0.010
PDZ and LIM domain protein 5 (PDLIM5)	A0A287BI36	Z-line	45	7	-0.402	< 0.001
PDZ and LIM domain protein 7 (PDLIM7)^4^	A0A4X1SEG0	Z-line	17	6	-0.741	< 0.001
*Structural*						
Desmin	P02540	Intermediate filament	63	24	-0.186	0.030
Nebulin	A0A287B5G8	Thin filament	55	21	0.142	0.029
Nebulin isoform 3	A0A480SKX8	Thin filament	58	2	-0.764	< 0.001
Obscurin^4^	A0A5G2QZ79	M-line	16	31	-0.228	0.006
Titin^4^	A0A5G2QM05	Thick filament	64	31	-0.346	< 0.001
Titin^4^	A0A4X1U902	Thick filament	57	13	-0.346	< 0.001
*Chaperone proteins*						
Heat shock protein β 1 (HSPB1)^4^	A0A5S6G3Y8	Multiple	24	8	-0.261	0.004
Heat shock protein β 6 (HSPB6)^4^	A0A4X1TIY7	Multiple	36	4	-0.302	0.048
*Metabolic*						
Alpha enolase^4^	A0A4X1W8R1	Sarcoplasm	41	11	0.269	0.017
Alpha-1,4 glucan phosphorylase	A0A4X1VBN9	Sarcoplasm	57	39	-0.710	< 0.001
ATP synthase F1 subunit delta	A0A4X1VPE5	Mitochondrial membrane	14	2	0.212	0.037
Beta enolase^4^	A0A4X1USV7	Sarcoplasm	53	14	-0.453	< 0.001
Creatine kinase M-type	Q5XLD3	M-line	55	18	-0.396	< 0.001
Fumarate hydratase, mitochondrial	A0A4X1TEE0	Mitochondrial matrix	11	4	-0.877	0.002
Glyceraldehyde-3-phosphate dehydrogenase	P00355	Sarcoplasm	77	10	-0.694	0.008
Glyceraldehyde-3-phosphate dehydrogenase (fragment)	G3CKJ2	Sarcoplasm	38	2	-0.685	0.004
Phosphoglycerate mutase 2	B5KJG2	Sarcoplasm	53	13	-0.235	0.023
Succinate–CoA ligase [ADP-forming] subunit beta, mitochondrial	F1RK10	Mitochondrial matrix	31	10	-0.274	0.004
*Uncategorized*						
14_3_3 domain-containing protein epsilon	A0A4X1U626	Sarcoplasm	67	12	-0.220	0.029
14_3_3 domain-containing protein gamma	A0A4X1UM41	Sarcoplasm	70	11	-0.155	0.037
CMP/dCMP-type deaminase domain-containing protein	A0A4X1SNG6		80	12	0.353	0.003
Cofilin 2^4^	B2CZR7		25	3	-0.511	0.037
Collagen alpha-2(VI) chain isoform 2C2	A0A480W6C8		16	12	0.140	0.031
Cytokeratin-1	F1SGG3		12	6	0.548	0.040
Elongation factor 1-α	A0A4X1SRH0		41	15	-0.207	0.003
Elongation factor 2	A0A287AWI9		12	7	-0.509	0.031
Histone H1.4	A0A480QWI4		22	5	0.442	0.017
Histone H2B	A0A287A7G8		41	6	0.161	0.029
Histone H4	P62802		57	6	0.450	0.003
Histone-lysine *N*-methyltransferase^4^	F1SVD5		39	17	-0.174	0.004
IF rod domain-containing protein	A0A4X1WBK5		9	4	0.567	0.029
Peptidase S1 domain-containing protein	A0A4X1V2S2		20	3	0.195	0.022
Perilipin 4	A0A287AV63		27	4	-0.607	0.016
Ubiquitin carboxyl-terminal hydrolase	F1RHF0		23	3	-0.815	0.027
WD repeat domain 1^4^	A0A4X1SGJ0		33	9	-0.364	0.046

^1^Accession number = Uniprot accession number.

^2^Sequence coverage = percent of the total number of identified amino acids/total number of amino acids.

^3^Log_2_ fold difference = group A/group D; positive number = greater in group A vs. D, negative number = lesser in group A vs. D.

^4^These proteins were initially labelled “Uncharacterized” or were labelled with a less commonly identified name. The FASTA sequence of the protein was used to match each protein to a more commonly recognized or accepted name using the UniProt BLAST feature.

### Thick and thin filament

The thick and thin filaments are the major components contributing to the basic and necessary organization of the sarcomere and myofibril for muscle contraction. The thick and thin filaments are comprised primarily of myosin and actin, respectively. Associated proteins aid in the binding of the thick and thin filaments through a highly organized series of reactions and protein conformational changes. In postmortem skeletal muscle following rigor mortis, permanent cross-links between the thick and thin filaments occur due to ATP depletion, resulting in permanently contracted sarcomeres compared to living skeletal muscle in the relaxed state. A weakening of the interaction of actin and myosin cross-links during the early postmortem period following ATP depletion has been associated with improved tenderness (Goll et al., 1997). The mechanisms explaining this proposed weakening are not fully explained; however, it has been established that myosin and actin are not preferential substrates for calpains in skeletal muscle (Dayton et al., 1976). Calpains have more specific protein targets, including desmin, troponin-T, titin, nebulin, and tropomyosin (Huff-Lonergan et al., 1996a; Lametsch et al., 2004).

The current study found a greater abundance (*P* < 0.05) of myosin-1, myosin-4, and actin in group A vs. D chops (Table 3). Although these are interesting observations, the differences in myosin heavy-chain isoforms are insufficient to conclude that muscle fiber type differed between these samples; but these observations, when in the context of other reports, demonstrate merit in additional investigations. Muscle fiber type using myosin heavy-chain as an indicator of commercial pork loins have not differed based on tenderness group (Carlson et al., 2017b), infection status, or residual feed intake (Outhouse et al., 2019). Lametsch et al. (2002) reported that some actin and myosin heavy-chain fragments in pork LD were generated during 0 to 48 h postmortem storage of pork LD. Smaller protein fragments of myosin heavy chain and actin are likely generated and released from the myofibrillar sub-proteome, as myosin heavy-chain and actin are substrates for various isoforms of cathepsins (Masanori et al., 1992) and caspases (Smuder et al., 2010).

Additional thick and thin filament proteins greater in abundance (*P* < 0.05) in group A vs. D chops included myosin-binding proteins C and H, myosin light chain 1, myosin regulatory light chain 2 and 9, tropomodulin 4, and troponin-T (Table 3). The myosin light chain and myosin regulatory light chains offer essential structural support of the myosin heavy-chain neck region while also providing some modulation of myosin ATPase activity (Sweeney et al., 1993; Sitbon et al., 2020). Myosin-binding proteins are associated with the thick filament (Gilbert et al., 1999), while tropomodulin (Weber et al., 1994) and troponin-T are localized on the thin filament.

Schulte et al. (2020) and Carlson et al. (2017a) reported one spot of myosin regulatory light chain 2 and one spot of myosin light chain 1, respectively, to be more abundant in the aged sarcoplasmic fraction from pork chops with a higher star probe value. The protein fraction from these studies differs from the current study, but proteolysis of myosin regulatory light chain 2 and myosin light chain 1 could result in the release of soluble fragments that appear in the aged sarcoplasmic proteome. Conversely, Anderson et al. (2012) demonstrated that calpain-1 rapidly degrades myosin light chain 1 from myofibers and that two spots of myosin light chain were more abundant in the sarcoplasmic fraction in beef. A previous study found a positive correlation between pork LD Warner-Bratzler shear force and myosin light chains 1 and 2 abundance at 3 d postmortem (Lametsch et al., 2003). Subsequent LC–MS/MS experiments with myofibrillar protein fractions have found troponin-T (Liu et al., 2021), myosin binding protein C, myosin light chain 1/3, and myosin regulatory light chain 2 (Sierra et al., 2021) to be differentially abundant based on quality or grouping in pork and beef. Two main explanations account for the observed variations in thick and thin filament proteins across groupings. The first is that there could be a diversity of muscle fiber types that explains the difference in association with the tenderness group. Alternatively, the second possibility is that postmortem aging with associated proteolysis, pH, and ionic strength results in a difference in protein properties, resulting in a change in solubility. Future experiments should be conducted to understand what protease systems and mechanisms ­contribute to these changes. Additionally, investigations should be conducted to define the extent to which fiber type contributes to the ­difference in the profile of these proteins in early postmortem and post-rigor muscle.

### Z-line or structural

The Z-line is the origin or starting point for numerous proteins that maintain the structure of and provide integrity to the sarcomere. A significant anchoring protein in the Z-line is α-actinin, which is a crucial structural and cross-linking protein. The thin filament is supported by a diverse range of proteins, such as titin, nebulin, actin, and myotilin, embedded in or extending from the Z-line. Some of these proteins also have multiple localizations or exist in multiple regions within the sarcomere, including titin, nebulin, and actin, all anchored in the Z-line but are primarily outside the Z-line. For example, titin is oriented with its N-terminus in the Z-line and C-terminus in the M-line, and nebulin ankers its C-terminus in the Z-line and extends the length of the thin filament (Meyer and Wright, 2013). Titin and nebulin maintain interactions with the thick and thin filament to support the overall sarcomere structure. Some binding partners of titin include α-actinin, myomesin, myosin-binding protein-C, and four-and-a-half LIM domain protein 1 (FHL1), and binding partners of nebulin include α-actinin, actin, tropomyosin, tropomodulin, and troponin isoforms (Kontrogianni-Konstantopoulos et al., 2009; Meyer and Wright, 2013). FHL1 localizes primarily to the Z-line/I-band and the M-line in mature skeletal muscle (Morgan and Madgwick, 1999; McGrath et al., 2006) and is involved in the regulation of skeletal muscle hypertrophy and sarcomere assembly (Cowling et al., 2008; Shathasivam et al., 2010).

Alpha-actinin 2, myotilin, myozenin 1, FHL1, LIM domain-binding protein 3 (LDB3), and PDZ and LIM domain protein 3 (PDLIM3), PDLIM5, and PDLIM7 were all less abundant (*P* < 0.05) in group A vs. group D in the current study (Table 3). Less α-actinin 2 in the myofibrillar fraction in group A is consistent with increased proteolysis in group A. Goll et al. (1991) showed that both calpain-1 and -2 catalyzed the release of α-actinin from myofibrils. Additionally, desmin, nebulin isoform 3, obscurin, and two isoforms of titin were all less abundant (*P* < 0.05) in group A vs. group D, and nebulin (UniProt: A0A287B5G8) was more abundant (*P* < 0.05) in group A vs. group D (Table 3). These observations are consistent with more proteolysis in group A. Desmin (Huff-Lonergan et al., 1996a; Cohen, 2020), titin, and nebulin (Huff-Lonergan et al., 1996a) are all substrates for calpains, and in pork, titin, desmin (Carlson et al., 2017b), and nebulin (Melody et al., 2004) degradation have been associated with a more tender pork product. Cohen (2020) proposed that calpain was specifically involved in desmin disassembly in living muscle. One-dimensional Western blot analysis of desmin confirmed the difference in desmin between groups A and D, specifically the ~55-kDa intact band of desmin (Figure 2A and B). The LC–MS/MS approach compared the intact and degradation products of desmin associated with the myofibrillar fraction because the entire myofibrillar fraction is digested with trypsin. These differences in methodologies and analysis between SDS-PAGE and LC-MS/MS are important to consider yet are complementary to building a comprehensive understanding of the specific molecular changes occurring in postmortem skeletal muscle.

Together, these differentially abundant proteins highlight the proteomic differences in the aged myofibrillar sub-­proteome of pork chops with different instrumental star probe values. All differentially abundant Z-line or structural proteins, except for one nebulin isoform (UniProt: A0A287B5G8), were significantly less in the more tender Group A chops. A lesser abundance of these myofibrillar proteins, which maintain key structural linkages in the Z-line from within and outside the myofibril, is associated with lower instrumental pork tenderness values. Previously, the degradation of the Z-line was not identified as a contributor to meat tenderness development, and instead, a disruption or weakening of thin filament connections to the Z-line and degradation of costameres and inter myofibril linkages were primarily responsible (Taylor et al., 1995). Taylor et al. (1995) stated that proteins other than α-actinin in the Z-line may partially contribute to meat tenderness development. The degradation of many proteins, such as titin, nebulin, and desmin, has been used as indicators of postmortem proteolysis and meat tenderness development. Calpain-1 incubation of at-death myofibrils resulted in similar protein degradation profiles of titin, nebulin, desmin, and troponin-T during postmortem aging (Huff-Lonergan et al., 1996a, 1996b). Baron et al. (2004) showed that calpain-1 and -2 degrade desmin primarily in the head and tail region, leaving the rod intact. Carlson et al. (2017a) proposed that the rod portion is the desmin product identified in the low ionic strength fraction in aged pork. Postmortem proteolysis and other biochemical changes are important for interpreting the results from the current study. Cellular changes of muscle during pre- and post-rigor can negatively impact calpain-1 and -2, including increased ionic strength, decreasing pH (from pH 7.0 to 6.0), exposure to hydrogen peroxide, association with calpastatin (Maddock et al., 2005; Carlin et al., 2006), and oxidation by lipid oxidation products (Zhai et al., 2023). These defined sources of variation and other undefined factors likely explain the differences in proteolysis of the aged myofibrillar sub-proteome from the current study.

These differentially abundant proteins in the current study add context to myofibrillar proteomic changes in postmortem tenderness development. The FHL1 and PDLIM proteins have been less characterized in postmortem skeletal muscle. However, it could be hypothesized that their lower abundance in the myofibrillar fraction from more tender pork chops is due to proteolysis by the calpain family of proteases. Alternatively, a binding partner of FHL1 and PDLIM proteins could be degraded, resulting in a structural change in the myofibrillar architecture and altering FHL1 and PDLIM proteins’ solubility, similar to α-actinin (Goll et al., 1991). More targeted experiments of FHL1 and PDLIM proteins and the other differentially abundant proteins are necessary to confirm that these proteins are substrates for calpain and that the differences result from proteolysis.

### Metabolic proteins

Metabolic enzymes are crucial in catalyzing many metabolic reactions to produce energy for the muscle cell to maintain normal cell function and contraction. Many of these enzymes exist in the soluble cytosol within the muscle cell. Metabolic enzymes maintain great influence over the biochemical changes occurring in postmortem skeletal muscle, and alterations of their activity and abundance, especially during the early postmortem period, can greatly influence meat quality. Creatine kinase M-type is known to localize on the M-line within the myofibril (Turner et al., 1973; Hornemann et al., 2003). The interaction of glycogen phosphorylase, phosphofructokinase, and other enzymes with a prepared F-actin-tropomyosin-troponin complex was enzyme dependent (Clarke and Masters, 1975) but suggests that a proportion of muscle enzymes that would be expected to be soluble under low-ionic conditions also interact to varying degrees with the myofibril.

In the present study, α-1,4 glucan phosphorylase, β-enolase, creatine kinase M-type, fumarate hydratase, glyceraldehyde-3-phosphate dehydrogenase, phosphoglycerate mutase 2, and succinate-CoA ligase subunit beta were all less abundant (*P* < 0.05) in group A vs. group D chops, while α-enolase and ATP synthase F1 subunit delta were greater in abundance (*P* < 0.05) in group A vs. group D chops (Table 3). These observations suggest that the solubility of these energy metabolic enzymes in postmortem muscle is important to the progression of pork tenderness development. Conversely, observation of these proteins that should be relatively soluble as more abundant in group D could mean that an environmental factor, like the rate of pH or temperature decline, could result in the denaturation and aggregation with the myofibrillar fraction. Interestingly, the myofibrillar fraction was extensively washed to remove soluble protein before protein solubilization. Therefore, these differentially abundant metabolic enzymes were likely strongly associated with the myofibril structure through covalent or non-covalent interactions.

Many metabolic proteins listed here are expected to be readily soluble in a low-ionic strength buffer and separated from myofibrils. Previous studies have also identified metabolic proteins associated with the myofibrillar protein fraction in early postmortem muscle by mass spectrometry approaches (Lametsch et al., 2011; Huang et al., 2012; Li et al., 2015; Liu et al., 2021, 2022). The observation that metabolic proteins or their products are more abundant in the myofibrillar sub-proteome of the chops from Group D likely indicates that their presence with this fraction is directly linked to the tougher phenotype. It is hypothesized that protein denaturation and precipitation of these metabolic proteins on or binding to the myofibril through a combination of elevated temperature and rapid pH decline early postmortem partly explains these observations in the current study (Yang et al., 2022). A greater abundance of glycogen phosphorylase was reported in the insoluble pellet from turkey pectoralis major samples with a lower pH (pH ≤ 5.8 at 15 min postmortem) than higher pH (pH > 6.0 at 15 min postmortem; Rathgeber et al., 1999). Other proteins, specifically calpain-1, become more associated with the myofibril during postmortem aging (Melody et al., 2004). Conditions that induce protein denaturation have been hypothesized to explain the association of protein conventionally soluble in the low-ionic strength fraction with the myofibril (Liu et al., 2016). Future, more targeted experiments should define the association of muscle enzymes that are conventionally thought to be soluble in low-ionic strength environments and the extent that these enzymes change and respond to various conditions in postmortem muscle, including decreasing pH and temperature and increasing ionic strength. These future studies would further define and better characterize the myofibrillar sub-proteome of aged pork and the extent that protein denaturation impacts the localization and solubility of these muscle enzymes.

## Conclusions

Pork loin quality, specifically tenderness, is important for consumer acceptance and eating satisfaction. Proteolysis of specific structural proteins within and outside the myofibril is known to occur and is associated with the development of pork tenderness, but there is a lack of comprehensive understanding of these molecular processes. The fractionation of the aged myofibrillar sub-proteome and analysis by LC-MS/MS in the current study provides a greater understanding of these changes. The current results support the hypothesis that proteolysis of many myofibrillar proteins contributed to the release of soluble protein and peptide fragments, resulting in their differential abundance in the aged myofibrillar sub-proteome. Future research must evaluate and relate earlier postmortem myofibrillar sub-proteome differences with meat tenderness. Additionally, more refined experiments should explore the extent that metabolic proteins are associated with the myofibril.

## Supplementary Material

skad327_suppl_Supplementary_Tables

## Abbreviations

AGC: automatic gain control
EDTA: ethylenediaminetetraacetic acid
EGTA: ethylenebis(oxyethylenenitroilo)tetraacetic acid
FDR: false discovery rate
HCD: higher energy collisional dissociation
LC–MS/MS: liquid chromatography–tandem mass spectrometry
SDS: sodium dodecyl sulfate
SDS-PAGE: sodium dodecyl sulfate-polyacrylamide gel electrophoresis
TEAM: triethyl ammonium bicarbonate
TMT: tandem mass tag
