# Supplementary material for: Distinct myofibrillar sub-proteomic profiles are associated with the instrumental texture of aged pork loin
Source: J Anim Sci. 2023 Sep 26;101:skad327. doi: 10.1093/jas/skad327 (PMC10629443; doi:10.1093/jas/skad327)
Supplement: skad327_suppl_Supplementary_Tables [file skad327_suppl_supplementary_tables.docx]

**Supporting Information for**

**Distinct myofibrillar sub-proteomic profiles are associated with the instrumental texture of aged pork loin**

Logan G. Johnson ^ƒ^, Chaoyu Zhai ^‡^, Edward M. Steadham ^ƒ^, Leah M. Reever ^§^,

Kenneth J. Prusa ^§^, Mahesh N. Nair ^#^, Elisabeth Huff-Lonergan ^ƒ^, and Steven M. Lonergan ^ƒ,1^

^ƒ^ Department of Animal Science, Iowa State University, Ames, Iowa 50011, USA

^‡^ Department of Animal Science, University of Connecticut, Storrs,

Connecticut 06269-4040, USA

^§^ Department of Food Science and Human Nutrition, Iowa State University,

Ames, Iowa 50011, USA

^#^ Department of Animal Sciences, Colorado State University, Fort Collins,

Colorado 80523, USA

^1^Corresponding author: slonerga@iastate.edu

**Supplementary Table 1.** Summary of pork quality traits from the initial population of 120 (*N* = 120) commercial pork loins.

| **Attribute** | **Range** | **Mean** | **Standard Deviation** |
| --- | --- | --- | --- |
| Star Probe, kg^1^ | 3.43 – 7.41 | 5.16 | 0.77 |
| 24 h pH | 5.51 – 6.01 | 5.68 | 0.10 |
| Aged pH | 5.62 – 6.07 | 5.78 | 0.09 |
| Loin Purge, % | 0.00 – 2.73 | 0.61 | 0.57 |
| Chop Purge, % | 0.06 – 1.86 | 0.74 | 0.34 |
| Moisture Content, % | 72.97 – 78.15 | 74.83 | 0.93 |
| Lipid Content, % | 0.83 – 4.86 | 2.03 | 0.76 |
| Marbling Score^2^ | 1.0 – 4.0 | 2.0 | 0.6 |
| Color Score^3^ | 2.0 – 4.5 | 3.2 | 0.6 |
| Aged *L** | 42.24 – 54.07 | 48.26 | 2.26 |
| Cook Loss, % | 15.57 – 27.74 | 22.49 | 2.45 |
| Tenderness | 4.0 – 10.0 | 6.8 | 1.1 |
| Chewiness | 1.0 – 6.0 | 3.2 | 0.9 |
| Juiciness | 5.0 – 9.0 | 6.6 | 0.9 |
| Flavor | 2.0 – 8.0 | 4.4 | 1.1 |
| Off Flavor | 1.0 – 5.0 | 1.7 | 0.8 |

^1^A 5-point star probe attachment fitted with an Instron was used to assess the force needed to compress a chop to 20% of its original height (Carlson et al., 2017b).

^2^National Pork Board standards, 10-point scale (1 = 1% intramuscular fat; 10 = 10% intramuscular fat).

^3^National Pork Board standards, 6-point scale (1 = pale pinkish gray/white; 6 = dark purplish red). ^4^Hunter L* determined with Minolta Chroma Meter with D65 light source, 50 mm aperture, and 0° observer.

^5^As determined by a trained panel (*N* = 4) using a 10-point category scale.

**Supplementary Table 2.** An overview of identified protein groups that were identified in the current study, contained at least 2 unique peptides, and were present in at least half (*n* ≥ 46) of the samples. These proteins were included in the analysis, but were not significantly (*P* ≥ 0.05) different between Group A and Group D.

| **Accession Number^1^** | **Description** | **Sequence Coverage^2^** | **Unique Peptides** | **A vs. D Log_2_ Fold Difference^3^** | **A vs. D Adjusted *P*-Value** |
| --- | --- | --- | --- | --- | --- |
| A0A4X1TUU7 | 4-alpha-glucanotransferase | 15 | 14 | 0.020 | 0.889 |
| B6V8C8 | 40S ribosomal protein S3a | 8 | 2 | -0.091 | 0.636 |
| A0A480VE77 | 60S ribosomal protein L8 (Fragment) | 12 | 2 | 0.058 | 0.711 |
| A0A480UES1 | 78 kDa glucose-regulated protein | 12 | 4 | 0.092 | 0.677 |
| K4HXJ4 | Actin (Fragment) | 39 | 2 | 0.128 | 0.641 |
| B2ZFN7 | Actin (Fragment) | 70 | 3 | -0.075 | 0.797 |
| A0A480VPN7 | Actin-depolymerizing factor (Fragment) | 18 | 8 | 0.114 | 0.371 |
| A0A480U0E1 | Actin, cytoplasmic 1 | 31 | 2 | 0.538 | 0.138 |
| A0A5G2RET9 | Actinin alpha 3 | 72 | 2 | -0.115 | 0.127 |
| A0A286ZQ79 | Adenylate kinase isoenzyme 1 | 30 | 7 | -0.074 | 0.684 |
| A0A4X1TTC3 | Adenylosuccinate synthetase isozyme 1 | 25 | 10 | -0.022 | 0.872 |
| F1RZQ6 | ADP/ATP translocase | 44 | 11 | 0.181 | 0.152 |
| A0A481CGG0 | Alpha-actinin-1 | 18 | 2 | 0.009 | 0.973 |
| A0A4X1TDZ5 | Alpha(B)-crystallin | 41 | 7 | -0.053 | 0.581 |
| A0A4X1SZL1 | AMP deaminase | 39 | 23 | -0.166 | 0.127 |
| A0A4X1VWL0 | AMP-binding domain-containing protein | 11 | 6 | 0.320 | 0.301 |
| K7GM40 | Apolipoprotein A-I | 27 | 6 | -0.271 | 0.164 |
| A0A480JGU8 | Aspartate aminotransferase | 24 | 9 | -0.137 | 0.229 |
| A0A5G2REF2 | Aspartate beta-hydroxylase | 16 | 3 | -0.055 | 0.567 |
| A0A8A0RU27 | ATP synthase F1 subunit alpha | 31 | 4 | 0.084 | 0.567 |
| A0A2C9F3A3 | ATP synthase peripheral stalk subunit OSCP | 28 | 5 | 0.107 | 0.306 |
| A0A5G2RAC9 | ATP synthase subunit b | 16 | 4 | 0.203 | 0.134 |
| A0A481D232 | ATP synthase subunit beta | 58 | 20 | 0.109 | 0.193 |
| A0A5G2QY12 | ATP synthase subunit d, mitochondrial | 45 | 7 | 0.174 | 0.088 |
| A0A287AHM1 | ATP synthase subunit gamma | 25 | 5 | -0.134 | 0.416 |
| A0A4X1UAD7 | ATP synthase-coupling factor 6, mitochondrial | 41 | 4 | 0.175 | 0.184 |
| A0A287B8N1 | ATP-dependent 6-phosphofructokinase | 55 | 32 | -0.105 | 0.067 |
| A0A4X1T6H5 | ATP-grasp domain-containing protein | 18 | 9 | -0.098 | 0.331 |
| A0A480VNC1 | B-cell receptor-associated protein | 10 | 3 | 0.046 | 0.844 |
| A0A287B1H6 | Bridging integrator 1 | 18 | 7 | -0.258 | 0.272 |
| A0A4X1VMP3 | BTB domain-containing protein | 43 | 21 | 0.173 | 0.105 |
| A0A5G2R940 | Calcium-transporting ATPase | 49 | 36 | -0.145 | 0.066 |
| A0A5S6HET4 | Calcium-transporting ATPase | 45 | 24 | -0.099 | 0.146 |
| A0A4X1V5U6 | Calcium/calmodulin-dependent protein kinase | 24 | 6 | 0.035 | 0.844 |
| F1RJW7 | Calsequestrin | 39 | 10 | -0.075 | 0.581 |
| Q5S1S4 | Carbonic anhydrase 3 | 44 | 8 | 0.062 | 0.641 |
| A0A287A2G4 | Caveolae associated protein 1 | 31 | 5 | -0.102 | 0.546 |
| A0A5G2R9Y3 | CBM20 domain-containing protein | 19 | 6 | -0.166 | 0.118 |
| A0A4X1VW06 | CCT-alpha | 6 | 3 | -0.078 | 0.674 |
| A0A480M2V3 | Collagen alpha-3(VI) chain isoform 4 | 20 | 37 | -0.005 | 0.935 |
| F1SLR1 | Complex I-19kD | 13 | 2 | -0.234 | 0.248 |
| Q2HYU1 | Creatine kinase | 26 | 7 | 0.355 | 0.084 |
| A0A481BJU4 | Cullin-associated NEDD8-dissociated protein 2 isoform 1 | 9 | 7 | 0.036 | 0.875 |
| F1S3W0 | Cytochrome b-c1 complex subunit 6 | 29 | 2 | 0.066 | 0.654 |
| A0A4X1U2M7 | Cytochrome b-c1 complex subunit 7 | 30 | 3 | 0.096 | 0.498 |
| A0A4X1TWD8 | Cytochrome b-c1 complex subunit Rieske, mitochondrial | 11 | 3 | 0.111 | 0.371 |
| A0A4X1UNI7 | Cytochrome c domain-containing protein | 15 | 3 | 0.066 | 0.771 |
| F1SJ34 | Cytochrome c oxidase polypeptide Va | 16 | 2 | 0.217 | 0.237 |
| A0A5G2QPD2 | Cytochrome c oxidase subunit 4 | 21 | 4 | 0.316 | 0.062 |
| Q5S3G4 | Cytochrome c oxidase subunit 5B, mitochondrial | 31 | 4 | 0.357 | 0.120 |
| A0A4X1SGJ1 | Decorin | 10 | 4 | -0.058 | 0.566 |
| A0A286ZKE7 | Dehydrogenase/reductase 7C | 26 | 5 | -0.050 | 0.903 |
| A0A4X1VRP4 | Dynamin-type G domain-containing protein | 37 | 23 | -0.139 | 0.059 |
| A0A1Y0DDA1 | Embryonic skeletal myosin heavy chain 3 (Fragment) | 28 | 5 | 0.073 | 0.714 |
| A0A480PE19 | Enoyl-CoA hydratase | 24 | 11 | 0.010 | 0.949 |
| A0PFK6 | F-actin-capping protein subunit alpha | 64 | 11 | 0.059 | 0.487 |
| A0A5K1U188 | F-actin-capping protein subunit beta | 42 | 12 | 0.089 | 0.079 |
| F1SMN5 | Filamin C | 41 | 80 | -0.092 | 0.053 |
| A0A4X1U0N5 | Fructose-bisphosphate aldolase | 49 | 2 | 0.038 | 0.774 |
| A0A5G2QD67 | Galectin | 51 | 8 | 0.080 | 0.487 |
| A0A4X1WCH7 | Glycerol-3-phosphate dehydrogenase [NAD(+)] | 28 | 10 | -0.051 | 0.564 |
| A0A4X1W016 | Glycogen [starch] synthase | 28 | 14 | -0.048 | 0.636 |
| A0A5G2QZY6 | GTP-binding nuclear protein Ran | 14 | 3 | -0.254 | 0.193 |
| P02067 | Hemoglobin subunit beta | 39 | 4 | 0.175 | 0.702 |
| F2Z5L5 | Histone H2A | 27 | 2 | 0.055 | 0.641 |
| I3LJY8 | Hydroxysteroid 17-beta dehydrogenase 12 | 6 | 2 | -0.008 | 0.972 |
| A0A287AEL2 | IF rod domain-containing protein | 16 | 5 | 0.539 | 0.056 |
| A0A4X1WBN0 | IF rod domain-containing protein | 8 | 2 | 0.839 | 0.064 |
| A0A4X1UKQ2 | IF rod domain-containing protein | 10 | 12 | -0.164 | 0.283 |
| A0A480YSS4 | Isocitrate dehydrogenase [NAD] subunit, mitochondrial | 17 | 4 | 0.005 | 0.972 |
| P33198 | Isocitrate dehydrogenase [NADP], mitochondrial (Fragment) | 43 | 16 | -0.121 | 0.091 |
| A0A287BK05 | Junctional sarcoplasmic reticulum protein 1 | 19 | 4 | 0.163 | 0.392 |
| F1RWK0 | Junctophilin | 4 | 2 | 0.289 | 0.567 |
| I3LDS3 | Keratin 10 | 15 | 7 | 0.542 | 0.099 |
| A0A480X8T8 | L-lactate dehydrogenase | 42 | 15 | -0.060 | 0.433 |
| A0A480NRZ3 | Lamin isoform A | 21 | 12 | -0.120 | 0.435 |
| A0A4X1TF57 | MARVEL domain-containing protein | 9 | 2 | 0.081 | 0.611 |
| A0A287AMM6 | MICOS complex subunit MIC60 | 7 | 4 | 0.114 | 0.636 |
| A0A4X1U7S9 | MIF4G domain-containing protein | 11 | 8 | 0.387 | 0.179 |
| A1XQS3 | Mitochondrial NDUFA4 | 33 | 3 | 0.149 | 0.137 |
| A0A1W6R2B4 | Mitsugumin-53 | 45 | 15 | -0.163 | 0.062 |
| A0A5G2QVC5 | Mono-ADP ribosylhydrolase 1 | 17 | 4 | 0.245 | 0.082 |
| A0A287AQJ5 | Multifunctional fusion protein | 21 | 19 | 0.143 | 0.111 |
| K4EJ64 | MYL3 | 81 | 12 | 0.136 | 0.134 |
| P02189 | Myoglobin | 36 | 4 | -0.286 | 0.063 |
| A0A5G2RCH7 | Myomesin 1 | 46 | 59 | 0.054 | 0.412 |
| A0A4X1STF9 | Myosin light chain kinase 2, skeletal/cardiac muscle | 8 | 4 | 0.086 | 0.723 |
| F1SS64 | Myosin-4 | 63 | 22 | 0.219 | 0.081 |
| P79293 | Myosin-7 | 63 | 36 | 0.140 | 0.082 |
| A0A287BPK4 | Myozenin 3 | 33 | 5 | -0.005 | 0.976 |
| A0A481BBQ7 | Nebulin isoform 3 | 54 | 2 | 0.066 | 0.208 |
| A0A480QS83 | Obscurin isoform IC | 5 | 2 | -0.057 | 0.859 |
| A0A4X1SNN9 | Phosphate carrier protein, mitochondrial | 6 | 2 | 0.191 | 0.324 |
| G0Z3A1 | Phosphoglucomutase 1 | 34 | 16 | 0.117 | 0.184 |
| F1RPH0 | Phosphoglycerate kinase | 54 | 17 | -0.049 | 0.636 |
| F1RPM0 | Phosphorylase b kinase regulatory subunit | 19 | 19 | -0.089 | 0.107 |
| A0A4X1TYG1 | Phosphorylase b kinase regulatory subunit | 23 | 21 | 0.014 | 0.818 |
| A0A024BTL2 | Phosphorylase kinase | 53 | 15 | -0.107 | 0.095 |
| A0A287B217 | Plectin | 6 | 21 | 0.010 | 0.949 |
| A0A5G2QDJ2 | Polyubiquitin-C | 45 | 3 | 0.178 | 0.418 |
| A0A5S6I5L2 | PRA1 family protein | 15 | 2 | 0.244 | 0.487 |
| A0A5G2RB75 | Receptor expression-enhancing protein | 10 | 2 | -0.053 | 0.702 |
| Q6IM77 | Reticulon | 12 | 3 | -0.058 | 0.564 |
| A0A4X1W6J5 | Reticulon | 30 | 5 | -0.029 | 0.818 |
| A0A4X1UFA1 | Ribosomal protein L19 | 14 | 2 | 0.575 | 0.120 |
| Q29104 | Ryanodine receptor 1 | 23 | 82 | 0.079 | 0.372 |
| A0A480TH91 | SH3 domain-binding glutamic acid-rich protein isoform a (Fragment) | 27 | 4 | 0.152 | 0.120 |
| A0A5G2Q7D7 | Succinate dehydrogenase [ubiquinone] flavoprotein subunit, mitochondrial | 28 | 9 | 0.068 | 0.421 |
| A0A5G2R4W7 | Succinate--CoA ligase [ADP/GDP-forming] subunit alpha, mitochondrial | 11 | 3 | -0.218 | 0.244 |
| A0A480SN35 | Titin isoform X6 | 61 | 223 | 0.076 | 0.125 |
| A0A4X1TLS9 | Triadin | 14 | 12 | -0.053 | 0.636 |
| A0A288CFT0 | Triosephosphate isomerase | 31 | 5 | 0.120 | 0.741 |
| A0A287BHM1 | Tropomyosin alpha-1 chain | 63 | 16 | 0.091 | 0.410 |
| A0A4X1VZV4 | Tropomyosin alpha-3 chain | 65 | 11 | 0.117 | 0.244 |
| P63317 | Troponin C, slow skeletal and cardiac muscles | 57 | 8 | 0.126 | 0.417 |
| B3VI70 | Troponin I | 40 | 7 | 0.086 | 0.272 |
| A0A5S6GU87 | Troponin T, slow skeletal muscle | 45 | 11 | 0.150 | 0.127 |
| A0A480NAY0 | Tubulin alpha chain | 51 | 3 | 0.088 | 0.392 |
| A0A480IZE0 | Tubulin alpha chain | 54 | 5 | 0.040 | 0.444 |
| A0A480TFQ0 | Tubulin beta chain | 48 | 3 | 0.049 | 0.611 |
| F2Z5B2 | Tubulin beta chain | 43 | 3 | 0.122 | 0.613 |
| F1SKM0 | Ubiquinol-cytochrome c reductase protein 1 | 43 | 13 | 0.066 | 0.392 |
| A0A4X1UEC7 | Uncharacterized protein | 56 | 4 | 0.298 | 0.060 |
| A0A5G2Q7R0 | Uncharacterized protein | 20 | 15 | 0.157 | 0.065 |
| A0A4X1VWS4 | Uncharacterized protein | 9 | 8 | 0.230 | 0.110 |
| A0A287AMA4 | Uncharacterized protein | 47 | 3 | 0.290 | 0.115 |
| A0A4X1VX90 | Uncharacterized protein | 52 | 12 | 0.136 | 0.129 |
| A0A4X1U3M3 | Uncharacterized protein | 81 | 10 | 0.170 | 0.156 |
| A0A4X1SIQ1 | Uncharacterized protein | 38 | 9 | 0.120 | 0.193 |
| A0A4X1V362 | Uncharacterized protein | 15 | 6 | -0.178 | 0.263 |
| A0A4X1VES0 | Uncharacterized protein | 30 | 12 | -0.111 | 0.272 |
| A0A4X1TKD2 | Uncharacterized protein | 25 | 2 | 0.197 | 0.272 |
| A0A4X1U197 | Uncharacterized protein | 18 | 4 | 0.180 | 0.435 |
| A0A5G2R6F6 | Uncharacterized protein | 35 | 5 | 0.103 | 0.437 |
| A0A4X1TY50 | Uncharacterized protein | 51 | 11 | 0.107 | 0.444 |
| A0A5G2QHR6 | Uncharacterized protein | 8 | 3 | 0.088 | 0.714 |
| A0A5G2RB89 | Uncharacterized protein | 14 | 5 | -0.062 | 0.723 |
| A0A4X1SJ49 | Uncharacterized protein | 55 | 49 | 0.021 | 0.797 |
| A0A4X1T308 | Uncharacterized protein | 63 | 3 | 0.023 | 0.815 |
| F1SM01 | Uncharacterized protein | 58 | 13 | 0.037 | 0.841 |
| A0A5G2R231 | Uncharacterized protein | 50 | 3 | 0.085 | 0.859 |
| A0A4X1VXU3 | Uncharacterized protein | 63 | 75 | 0.005 | 0.949 |
| A0A4X1W9B9 | Uncharacterized protein | 25 | 2 | 0.019 | 0.949 |
| A0A287BBB5 | VAMP associated protein A | 8 | 2 | -0.147 | 0.087 |
| A0A4X1V5C3 | Very-long-chain enoyl-CoA reductase | 3 | 2 | 0.066 | 0.636 |
| A0A4X1UV26 | Voltage-dependent anion-selective channel protein 1 | 36 | 10 | 0.135 | 0.146 |
| Q9MZ15 | Voltage-dependent anion-selective channel protein 2 | 30 | 7 | 0.162 | 0.199 |
| Q29380 | Voltage-dependent anion-selective channel protein 3 | 9 | 2 | 0.103 | 0.582 |

^1^Accession Number = Uniprot Accession Number.

^2^Sequence Coverage = Percent of the total number of identified amino acids/total number of amino acids.

^3^Log_2_ Fold Difference = Group A/Group D; Positive number = Greater in Group A vs. D, Negative number = Lesser in Group A vs. D.
